# Rebalancing the Gut: Glucagon-Like Peptide-1 Agonists as a Strategy for Obesity and Metabolic Health

**DOI:** 10.7759/cureus.64738

**Published:** 2024-07-17

**Authors:** Kanwarmandeep Singh, Smriti K Aulakh, Gurkamal Singh Nijjar, Sumerjit Singh, Ajay Pal Singh Sandhu, Shivansh Luthra, Fnu Tanvir, Yasmeen Kaur, Abhinandan Singla, Meet Sirjana Kaur

**Affiliations:** 1 Internal Medicine, Government Medical College, Amritsar, IND; 2 Internal Medicine, Sri Guru Ram Das University of Health Sciences and Research, Amritsar, IND; 3 Internal Medicine, Government Medical College, Patiala, IND

**Keywords:** gut-brain axis communication, short-chain fatty acids, weight management drugs, metabolic health, gut microbiome, gut microbiota dysbiosis, obesity treatment, glp-1 agonists

## Abstract

Obesity significantly impacts gut microbial composition, exacerbating metabolic dysfunction and weight gain. Traditional treatment methods often fall short, underscoring the need for innovative approaches. Glucagon-like peptide-1 (GLP-1) agonists have emerged as promising agents in obesity management, demonstrating significant potential in modulating gut microbiota. These agents promote beneficial bacterial populations, such as *Bacteroides*,* Lactobacillus*,* and Bifidobacterium*, while reducing harmful species like *Enterobacteriaceae*. By influencing gut microbiota composition, GLP-1 agonists enhance gut barrier integrity, reducing permeability and systemic inflammation, which are hallmarks of metabolic dysfunction in obesity. Additionally, GLP-1 agonists improve metabolic functions by increasing the production of short-chain fatty acids like butyrate, propionate, and acetate, which serve as energy sources for colonocytes, modulate immune responses, and enhance the production of gut hormones that regulate appetite and glucose homeostasis. By increasing microbial diversity, GLP-1 agonists create a more resilient gut microbiome capable of resisting pathogenic invasions and maintaining metabolic balance. Thus, by shifting the gut microbiota toward a healthier profile, GLP-1 agonists help disrupt the vicious cycle of obesity-induced gut dysbiosis and inflammation. This review highlights the intricate relationship between obesity, gut microbiota, and GLP-1 agonists, providing valuable insights into their combined role in effective obesity treatment and metabolic health enhancement.

## Introduction and background

Obesity, affecting one in eight individuals globally [1], presents a critical public health challenge. As defined by the WHO using the BMI, obesity classifications start with a BMI of 25.0-29.9 for overweight individuals [2]. A BMI between 30.0 and 34.9 indicates Obesity Class 1, 35.0-39.9 falls into Obesity Class 2, and a BMI of 40 or above is considered Obesity Class 3, marking an extremely high risk of developing serious health complications [2]. Since 1990, the global prevalence of obesity among adults has more than doubled [1]. By 2022, 43% of adults were classified as overweight, and 16% were obese [1]. Among children and adolescents aged 5-19 years, the prevalence of overweight increased from 8% in 1990 to 20% in 2022, with 8% living with obesity [1]. The consequences of obesity are extensive and severe, significantly increasing the risk of chronic diseases such as type 2 diabetes, cardiovascular conditions like hypertension and coronary artery disease [3], and various cancers, including those of the breast, colon, and endometrium [4]. Furthermore, obesity negatively affects bone health, elevating the likelihood of osteoarthritis and fractures [5], and it can lead to reproductive issues like polycystic ovary syndrome in women and reduced sperm quality in men [6]. Beyond these physical health impacts, obesity also places substantial economic burdens on societies through increased healthcare costs and diminished productivity, as evidenced by higher rates of absenteeism and reduced work efficiency among those affected [7].

In addition to its complex health and economic repercussions, there is one particular physiological effect of obesity that needs detailed discussion. That is its significant influence on the gut microbiome, which is the complex community of microorganisms residing in the intestinal tract. These microbes play essential roles in digesting fiber, synthesizing vitamins, stimulating the immune system, and protecting against pathogens. Moreover, gut microbes produce by-products that have significant effects on the body, both locally and systemically. Short-chain fatty acids (SCFAs) produced by gut bacteria are suggested to have anti-inflammatory and tumor-suppressive effects in the gut [8]. SCFAs have also been shown to stimulate the production of tight junction proteins, resulting in increased blood-brain barrier integrity [9]. These microbes also produce multiple vitamins, including thiamine, riboflavin, biotin, cobalamin, and vitamin K [10]. Thus, a balanced and diverse gut microbiota is crucial for overall health, impacting everything from nutrient absorption and energy regulation to immune function and mood [11]. However, obesity disrupts this balance, notably increasing the ratio of *Firmicutes *to *Bacteroidetes*. This shift enhances the body’s ability to harvest energy from the diet, leading to further weight gain and metabolic imbalance. The resulting microbial imbalance reduces overall microbial diversity and alters the production of SCFAs [12]. Additionally, obesity can compromise the integrity of the intestinal barrier, increasing its permeability [13]. This allows bacterial components to enter the bloodstream, triggering systemic inflammation and exacerbating metabolic dysfunction, thereby creating a vicious cycle that perpetuates the condition and complicates its management [13].

Management strategies for obesity encompass a range of approaches, from lifestyle modifications such as diet and exercise to medical interventions including pharmacotherapy and bariatric surgery. Lifestyle changes often serve as the first line of defense, encouraging healthier eating patterns and increased physical activity. For individuals whose adjustments prove insufficient, specific pharmacological treatments can provide additional support. Commonly prescribed medications for obesity include phentermine, orlistat, and newer agents like glucagon-like peptide-1 (GLP-1) agonists [14].

GLP-1 agonists represent a significant advancement, particularly due to their dual role in managing diabetes and obesity [15]. These drugs mimic the action of the GLP-1 hormone, which is naturally released after eating and plays a crucial role in regulating blood glucose levels and satiety [16]. By activating GLP-1 receptors (GLP-1Rs) in the brain, these agonists reduce caloric intake through enhanced feelings of fullness and slowed gastric emptying, thereby moderating nutrient absorption and stabilizing blood glucose levels [17]. Importantly, GLP-1 agonists also exert beneficial effects on the gut microbiota. They promote the growth of beneficial bacterial populations such as *Lactobacillus *and *Bifidobacterium *while reducing harmful species like *Enterobacteriaceae *[18]. This advantageous adjustment of the gut microbiota strengthens intestinal barrier integrity, reduces systemic inflammation, and enhances metabolic functions. Additionally, these changes in the microbiota can affect drug pharmacokinetics, modulate immune responses, and influence lipid metabolism [19].

In this paper, we investigate the intricate relationships between obesity, the gut microbiome, and GLP-1 agonists to better understand their roles in the obesity crisis. Our focus is on the mechanisms by which GLP-1 agonists influence the gut microbiota. By analyzing these interactions, this discussion aims to shed light on the potential of GLP-1 agonists through their modulation of the gut microbiota, serving as key components in effective obesity treatment.

## Review

GLP-1 agonists, obesity, and gut microbiota interactions

Obesity is associated with increased adipose tissue distribution in the body, which is a metabolically active organ that secretes a range of hormones and cytokines. In non-obese individuals, it primarily releases anti-inflammatory factors such as IL-1, IL-4, IL-10, and IL-13 receptor antagonists, transforming growth factor beta, and adiponectin [20], whereas in obese individuals, the nature of these secretions shifts toward pro-inflammatory factors like angiotensin II, IL-6, tumor necrosis factor alpha, leptin, and plasminogen activator inhibitor 1 [20]. These pro-inflammatory cytokines contribute to inhibiting β-cell proliferation and the release of insulin in response to glucose [21], leading to a state of chronic low-grade inflammation that affects various organ systems, including the gut. This state of inflammation is linked with alterations in gut microbial composition, which in turn affects the body’s energy extraction processes from food. In obese individuals, there is a noted increase in energy extraction due to changes in the gut microbiota [22]. A study conducted by Palmas et al. [23] revealed that obese individuals tend to have a higher presence of bacteria from the *Firmicutes *phylum, like *Blautia hydrogenotrophica *and *Ruminococcus obeum*, while lean individuals generally have more from the *Bacteroidetes *phylum, such as *Bacteroides thetaiotaomicron*. This imbalance results in a higher *Firmicutes *to *Bacteroidetes *ratio in individuals with obesity [24], which is associated with increased overall adiposity, dyslipidemia, and impaired glucose regulation [25].

GLP-1 agonists are widely used for the treatment of obesity [26], which exert their effect by binding to GLP-1Rs. GLP-1Rs are variably distributed throughout the body. They are highly concentrated in the duodenum’s Brunner’s glands and found at lower levels in the stomach’s muscularis externa and the intestinal myenteric plexus neurons [27]. Outside the gut, GLP-1Rs are prevalent in the CNS, specifically in areas such as the hippocampus, neocortex, hypothalamus, spinal cord, and cerebellum [28]. The primary signaling pathway of GLP-1Rs is initiated by the activation of the Gαs protein [29]. It enhances insulin secretion from pancreatic beta cells [30], moderates postprandial glucose by slowing gastric motility and reducing gastric emptying [31], and contributes to appetite regulation and weight management through feelings of satiety and fullness [32]. Activation of hypothalamic and brainstem GLP-1Rs triggers signaling that increases activity in appetite-suppressing neurons and inhibits appetite-promoting pathways involving neuropeptide Y and agouti-related peptide. Simultaneously, it enhances satiety pathways mediated by pro-opiomelanocortin and corticotropin-releasing hormone, leading to reduced appetite and food intake [33,34]. This is one of the mechanisms by which GLP-1 agonists exert their therapeutic effects on obesity and metabolic disorders.

**Figure 1:**
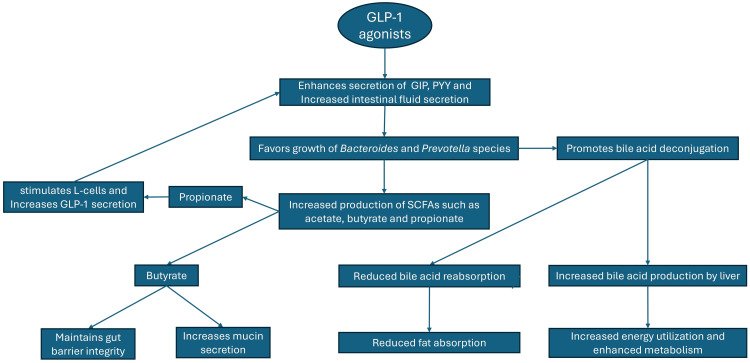
Impact of GLP-1 agonists on gut microbiota and metabolism The figure shows how GLP-1 agonists favor the growth of *Bacteroides *and *Prevotella *species and enhance metabolism through increased energy utilization and bile acid production. GIP, glucose-dependent insulinotropic polypeptide; GLP-1, glucagon-like peptide-1; PYY, peptide YY; SCFA, short-chain fatty acid Image credits: Kanwarmandeep Singh and Gurkamal Singh Nijjar

GLP-1 agonists also impact gut microbiota composition. This interaction with the gut microbiota represents an additional mechanism through which these drugs exert their therapeutic effects on obesity. They can modify the gut microbiota, potentially reversing the detrimental shifts induced by obesity and aiding in its treatment. In the following sections, we will discuss three primary ways in which GLP-1 agonists can contribute to the treatment of obesity through their effect on the gut microbiota. These pathways are illustrated and summarized in Figure 1.

SCFA production

GLP-1 agonists significantly enhance the secretion of gut hormones like peptide YY (PYY) and glucose-dependent insulinotropic polypeptide [35]. This elevation in hormone levels increases intestinal fluid secretion, creating a nutrient-rich environment that is particularly beneficial for specific gut microbes, such as *Bacteroidetes *and *Prevotella *[36]. This selective advantage allows these bacteria to flourish and excel at fermenting dietary fibers like inulin and resistant starches. Their activities lead to increased production of SCFAs such as acetate, propionate, and butyrate. Propionate, in particular, directly boosts GLP-1 secretion from L-cells, establishing a positive feedback loop that enhances its effects. SCFAs also activate G protein-coupled receptor 43 on L-cells and other gut cells, contributing to further GLP-1 release [35]. Butyrate is crucial for gut barrier integrity because it serves as the main energy source for colonocytes. It stimulates the production of tight junction proteins to prevent harmful substances from passing into the bloodstream. It also promotes the secretion of mucin, a protein that forms a protective mucus layer on the intestinal lining. It also has anti-inflammatory properties that help reduce inflammation and repair damage in the gut lining, thereby maintaining a healthy and functional intestinal barrier [37]. Additionally, SCFAs modulate immune function and reduce inflammation in the gut [38]. These effects combined influence appetite regulation, insulin sensitivity, and fat storage, showcasing the potential of GLP-1 agonists in weight management. However, due to individual differences in gut microbiota composition, the treatment efficacy of these agonists can vary among individuals [39].

Gut-brain axis regulation

Gut-brain axis regulation refers to the complex communication network between the gut microbiota and the CNS, particularly the brain. This involves hormonal, neural, and immune signaling pathways [40]. In the hormonal pathway, gut microbes produce metabolites like serotonin and gamma-aminobutyric acid that act as hormones, influencing brain functions such as appetite, mood, and cognition [41]. In the neural pathway, the vagus nerve transmits signals related to gut motility, appetite, and stress responses [42]. In the immune pathway, the gut’s immune system interacts with the CNS through the release of cytokines and other immune signaling molecules, impacting mood, behavior, and neuroinflammation [43].

Obesity establishes a damaging cycle in the gut, largely driven by high-fat, high-sugar diets that promote the growth of *Firmicutes *bacteria and reduce the growth of *Bacteroides *[44]. These bacteria produce inflammatory molecules that damage the gut barrier, causing increased gut permeability to stimulate systemic inflammation [13]. This inflammation disrupts gut-brain communication through the vagus nerve and hormonal pathways, leading to weakened satiety signals and increased cravings for unhealthy foods, which complicates weight management [45]. It also contributes to mood swings and emotional eating due to altered mood regulation [45]. Chronic inflammation linked to obesity raises stress hormones like cortisol [46], which further disrupts the gut microbiome and diminishes SCFA production, creating a feedback loop that promotes unhealthy eating habits and weight gain.

GLP-1 agonists can play a pivotal role in disrupting the vicious cycle of obesity by supporting the growth of beneficial gut bacteria, which enhances gut mucosal integrity [47] and optimizes nutrient availability [48]. By altering this composition, they help increase the production of SCFAs, which strengthens satiety signals and reduces cravings for unhealthy foods, ultimately supporting healthier weight management [49].

Bile acid metabolism

Obesity significantly alters bile acid metabolism through several mechanisms. It often causes increased bile acid production in the liver, likely compensating for the higher dietary fat intake associated with obesity [50]. GLP-1 agonists can reshape the gut microbiome by promoting beneficial bacteria such as *Akkermansia muciniphila*, *Verrucomicrobia*, and *Bacteroidetes *[36] that indirectly affect bile acid metabolism. Gut bacteria alter the structure of bile acids through deconjugation, a process that has a significant impact on bile acid reabsorption and the efficiency of fat absorption. GLP-1 agonists promote the growth of bacteria that enhance deconjugation [51]. Once deconjugated, bile acids are more effectively reabsorbed in the intestines and returned to the liver for reuse. Consequently, with fewer bile acids remaining in the intestines to break down new fats, the overall absorption of dietary fats is reduced. This reduction in fat absorption means fewer calories from fats are taken in by the body, potentially decreasing overall calorie intake and aiding in weight management. In response, the liver increases its production of bile acids. This increased production enhances metabolism and further increases energy utilization.

Discussion

In the complex relationship between microbial composition, dietary influences, and metabolic and inflammatory pathways, GLP-1 agonists have emerged as a promising modality for reshaping gut microbial diversity toward a healthier profile. GLP-1 agonists demonstrate a significant ability to modulate the gut microbiota composition, which represents a promising strategy for addressing obesity and its associated inflammatory environment and offers hope for newer, more effective therapeutic strategies.

Ley et al. discovered that obese individuals, even after significant weight loss, did not fully normalize the ratio of *Firmicutes *to *Bacteroidetes *observed in lean individuals [52]. These observations highlight the impact of dietary factors on gut microbiota composition in the context of obesity. Expanding on this, Feng et al. demonstrated a compelling correlation between the abundance of *Faecalibacterium prausnitzii *and fasting blood glucose levels among patients receiving GLP-1 agonist therapy, implicating gut microbes in the intricate regulation of blood glucose dynamics [53]. Similarly, Stenman et al. revealed a positive association between *Bifidobacterium *levels and enhanced insulin sensitivity, suggesting *Bifidobacterium *is a potential mediator of metabolic health, likely through its facilitation of GLP-1 production [54]. Yang et al. demonstrated that *A. muciniphila *supplementation led to elevated levels of GLP-1 and PYY, both of which suppress appetite and improve obesity [55]. Additionally, Depommier et al. found that *A. muciniphila *supplementation in diet-induced obese mice resulted in reductions in body weight gain and fat mass, along with an increase in fecal caloric content [56]. These findings highlight the intricate symbiosis between the gut microbiota and the therapeutic efficacy of GLP-1 agonists in managing metabolic health.

Zhang et al. observed that in diabetic male rats, liraglutide was associated with more SCFA-producing bacteria, and their gut microbiota became more varied in general [57]. This helped reduce inflammation and improve obesity. They also suggested the same reason for drugs like liraglutide being good for gut bacteria and overall health. Ying et al. observed similar results and noticed that liraglutide led to more diversity in two types of bacteria, *Bacteroidetes *and *Proteobacteria *[58]. These bacteria are linked to more SCFA production, which contributes to reduced inflammation and helps with weight loss [35]. These studies emphasize the effect of liraglutide on specific gut bacteria. Moreover, investigations by Tsai et al. reinforced the association between GLP-1 agonists and specific gut microbial alterations and explained their role in promoting anti-inflammatory and anti-obesity effects through modulation of the gut microbiota [39].

Beyond metabolic regulation, Hunt et al. provided evidence for the anti-inflammatory properties of GLP-1 agonists in reducing obesity-related inflammation, thereby expanding the therapeutic range of these agents [59]. Collectively, these findings signify the multifaceted impact of GLP-1 agonists on gut microbiota composition and metabolic health, offering promising ways for the development of targeted therapeutic interventions for obesity and related metabolic disorders. To further elucidate these relationships, Table 1 summarizes various studies that investigate the interaction between GLP-1 agonists, obesity, and gut microbiota.

**Table 1:** Studies demonstrating the interaction between GLP-1 agonists, obesity, and the gut microbiota This table summarizes various studies that investigate the relationship between GLP-1 agonists, obesity, and the gut microbiota. It highlights the impact of GLP-1 agonists on gut microbiota composition, metabolic health, and inflammation, providing insights into their potential mechanisms and therapeutic benefits in obesity management. GLP-1, glucagon-like peptide-1; GLP-1R, glucagon-like peptide-1 receptor; PYY, peptide YY; SCFA, short-chain fatty acid

Study design	Type of study	Result	Significance
Tsai et al. (2021) [39]	Observational	*Roseburia* species were associated with decreased obesity and dyslipidemia, potentially enhancing GLP-1 responsiveness. *Prevotella*/*Bacteroides *ratio was associated with obesity, with *Prevotella *potentially contributing to insulin resistance.	Highlights the association between specific bacterial taxa and metabolic health markers
Ley et al. (2005) [52]	Observational	Decreased *Firmicutes* and increased *Bacteroidetes *in obese individuals on low-fat or low-carbohydrate diets, but levels did not fully normalize compared to lean individuals.	Indicates the impact of diet on gut microbiota composition in obesity
Feng et al. (2014) [53]	Observational	*Faecalibacterium prausnitzii* showed a significant negative correlation with fasting blood glucose levels in GLP-1 agonist-treated patients, implicating gut microbes in blood glucose regulation.	Suggests a link between gut microbiota and GLP-1 agonist efficacy in managing blood glucose levels
Stenman et al. (2015) [54]	Observational	*Bifidobacterium *was positively correlated with increased insulin sensitivity, likely due to enhanced GLP-1 production.	Indicates the potential of *Bifidobacterium *as a mediator of metabolic health
Yang et al. (2020) [55]	Interventional	Supplementation with *Akkermansia muciniphila *increased GLP-1 and PYY levels, contributing to appetite suppression and weight reduction.	Highlights the potential of *A. muciniphila *as a therapeutic target for obesity
Depommier et al. (2020) [56]	Experimental	*A. muciniphila *supplementation in diet-induced obese mice reduced body weight gain, total adiposity index, and fat mass gain, alongside increased fecal caloric content.	Indicates the potential of *Bifidobacterium *as a mediator of metabolic health
Zhang et al. (2018) [57]	Observational	Liraglutide was associated with increased SCFA-producing bacteria and a more diverse microbiota, potentially promoting anti-inflammatory and anti-obesity effects.	Suggests a mechanism for the beneficial effects of GLP-1 agonists on gut microbiota and metabolic health
Ying et al. (2023) [58]	Observational	Liraglutide was found to be associated with an increased diversity of *Bacteroidetes *and *Proteobacteria*, which are associated with increased SCFA production and anti-inflammatory effects, as well as anti-obesity effects.	Highlights the association between liraglutide and specific gut microbial changes
Hunt et al. (2021) [59]	Experimental	Exendin-4, a GLP-1-like peptide, reduced pro-inflammatory cytokine production and decreased immune response in the gut.	Indicates the anti-inflammatory potential of GLP-1 agonists in obesity-related inflammation
Hippe et al. (2016) [60]	Observational	Reduction in *F. prausnitzii *was associated with increased hemoglobin A1c values, suggesting a role in metabolic health.	Highlights the potential significance of *F. prausnitzii *in glucose regulation
Dong et al. (2022) [61]	Observational	Subjects with visceral obesity had significantly decreased levels of *Bifidobacterium*.	Suggests a potential role of *Bifidobacterium *in metabolic health
Turnbaugh et al. (2008) [62]	Experimental	A lard-based saturated fat diet in rodents was associated with an increased ratio of *Firmicutes*/*Bacteroidetes *along with an absolute decrease in *Bacteroidetes *levels.	Demonstrates the influence of dietary fats on gut microbiota composition
Li et al. (2016) [63]	Experimental	Palm oil was found to be associated with an increased *Firmicutes/Bacteroidetes *ratio.	Indicates the impact of specific dietary components on gut microbiota composition
Ryu et al. (2023) [64]	Observational	Liraglutide has been linked with an increased abundance of *Bacteroidetes *and *Proteobacteria *in the gut, correlating with elevated production of SCFAs, anti-inflammatory properties, and anti-obesity benefits.	Further supports the association between Liraglutide and specific gut microbial changes
Wärnberg et al. (2004) [65]	Observational	Increasing BMI was correlated with elevated IL-6 levels, indicating a pro-inflammatory state in obese individuals.	Highlights the association between obesity and systemic inflammation

## Conclusions

GLP-1 agonists have emerged as a promising therapeutic option for obesity by significantly influencing gut microbiota composition and function. These agents enhance the growth of beneficial bacteria, such as *Bacteroidetes*, and promote the production of SCFAs, which are crucial for gut health. Studies demonstrate that GLP-1 agonists improve gut barrier integrity, reduce systemic inflammation, and modulate metabolic pathways, leading to better glucose regulation and weight management. The positive feedback loop between SCFA production and GLP-1 secretion amplifies these beneficial effects. The ability of GLP-1 agonists to shift the microbial balance toward a healthier profile underscores their potential to disrupt the harmful cycle of obesity-induced gut dysbiosis and inflammation. Personalized treatment strategies considering individual gut microbiota variations may further enhance the efficacy of GLP-1 agonists. This multifaceted impact on gut microbiota and metabolic health positions GLP-1 agonists as a critical component in developing comprehensive and effective obesity treatments.
